# Rapid degradation of methylene blue in a novel heterogeneous Fe_3_O_4_ @rGO@TiO_2_-catalyzed photo-Fenton system

**DOI:** 10.1038/srep10632

**Published:** 2015-05-22

**Authors:** Xiaoling Yang, Wei Chen, Jianfei Huang, Ying Zhou, Yihua Zhu, Chunzhong Li

**Affiliations:** 1 East China University of Science and Technology, Key Laboratory for Ultrafine Materials of Ministry of Education, School of Materials Science and Engineering, Shanghai 200237, China

## Abstract

Herein, a ternary nanocomposite with TiO_2_ nanoparticles anchored on reduced graphene oxide (rGO)-encapsulated Fe_3_O_4_ spheres (Fe_3_O_4_@rGO@TiO_2_) is presented as a high efficient heterogeneous catalyst for photo-Fenton degradation of recalcitrant pollutants under neutral pH. Fe_3_O_4_@rGO@TiO_2_ was synthesized by depositing TiO_2_ nanoparticles on the surface of the Fe_3_O_4_ spheres wrapped by graphene oxide (GO) which was obtained by an electrostatic layer-by-layer method. This as-prepared catalyst reflected good ferromagnetism and superior stability which makes it convenient to be separated and recycled. Due to the synergic effects between the different components composed the catalyst, swift reduction of Fe^3+^ can be achieved to regenerate Fe^2+^. Fe_3_O_4_@rGO@TiO_2_ exhibited enhancing catalytic activity for the degradation of azo-dyes compared with Fe_3_O_4_, Fe_3_O_4_@SiO_2_@TiO_2_ or SiO_2_@rGO@TiO_2_, further conforming the rapid redox reaction between Fe^2+^ and Fe^3+^. All these merits indicate that the composite catalyst possesses great potential for visible-light driven destruction of organic compounds.

Recently, the ever-growing emission of dye wastewater from various industries such as textiles, printing, food and cosmetics has become a major threat to human and ecology owing to the toxicity and non-biodegradability^1^,^2^,^3^. Many methods such as adsorption, flocculation and chemical oxidation have been used to remove the persistent dyes released into aquatic environment. Compared with other methods, one of the chemical oxidation methods called Fenton reaction has attracted intensive attention due to its capability to destruct refractory organic pollutants and turn them into low-molecule-weight inorganic compounds^4^,^5^,^6^,^7^. Hydroxyl radicals (·OH), as the key intermediates of Fenton process, holds highly oxidative potential and can attack almost all the organic compounds in a non-selective way, leading finally to the mineral end-products^7^. However, high activity of the reaction between the ferrous ions and hydrogen peroxide are always limited to a pH value around 3. Moreover, homogeneous Fenton systems can generate a great deal of iron sludge which may cause secondary pollution and increase the operating costs^8^,^9^,^10^.

In order to deal with these issues, heterogeneous Fenton-like reactions based on solid catalysts have shown great promise to replace the iron salt-based homogeneous Fenton systems^11^,^12^. Currently, great attention has been paid to the heterogeneous Fenton-like catalysts such as zero-valent iron, iron-based materials and iron-containing materials^13^,^14^,^15^. Among them, magnetite (Fe_3_O_4_) nanoparticles have attracted considerable research interests because of its unique properties, including decent magnetic, electric, catalytic properties, biocompatibility and low toxicity. Nevertheless, the nano-scaled magnetite particles have a tendency to aggregate to form larger particles, reducing the original large specific surface area and dispersibility, which will finally undermine the catalytic activity. Therefore, it is essential to immobilize these nanoparticles onto supports or encapsulate them within protective layer to maintain their unique performances^16^,^17^. Besides, the catalytic efficiency of the Fe_3_O_4_ nanoparticles await further promotion considering the relatively low conversion rate between the Fe^2+^ and the Fe^3+^ when they are used as the Fenton catalysts. Rationally, hybridization of Fe_3_O_4_ with a speeding-up component is desirable to boost the conversion between Fe^3+^ and Fe^2+^. Herein, we choose TiO_2_ to fulfill this task. When the electrons in TiO_2_ are irradiated by UV-visible light, they can be excited from the valance band to the conduction band to generate electron-hole pairs^18^. The photoexcited electrons can quickly transport to Fe^3+^, accelerating the redox transformation between Fe(III) and Fe(II). Meanwhile, the holes generated simultaneously can also react with H_2_O to produce highly oxidative hydroxyl radicals. The as-generated radicals, together with the holes equipped with high oxidative potential, can mineralize the persistent organic pollutants nonselectively^19^. However, there are two aspects need to be concerned when utilizing TiO_2_ as a photo-assisted cocatalyst. First, the high energy of irradiating photons required as a result of the wide band gap (3.20 eV) of the anatase TiO_2_ restricts its photoactivity to the narrow light-response range of ultraviolet accounting for only about 3–5% of total sunlight. Second, the photo-induced electron-hole pairs suffer from high recombination rate, so the reactivity of the electron-hole pairs will rapidly diminish^20^,^21^. In order to solve these problems and strengthen the combination between TiO_2_ and Fe_3_O_4_, an effective interlayer should be introduced.

In recent decades, graphene has attracted tremendous attention due to its excellent electronic properties and promising applications in various fields^22^,^23^. In this work, we selected it as the support material to enhance the synergistic effect between TiO_2_ and Fe_3_O_4_, and configured a ternary-composite design of Fenton catalyst. The advantages of this design can be concluded as follows. First, when TiO_2_ was combined with graphene, the electrons in the valance band of TiO_2_ can be excited to conduction band under the irradiation of visible light^24^,^25^,^26^,^27^,^28^,^29^,^30^ and the existence of graphene will prolong the lifetime of the photoexcited electron-hole pairs and thus contributing to higher possibility of successful transfer of the electrons from the TiO_2_ to Fe^3+^/Fe^2+^ redox pair. Second, the graphene-wrapped Fe_3_O_4_ has better dispensability in aqueous medium. Besides, the presence of various oxygen-containing groups such as carboxyl, epoxides, alcohols, lactols^31^ on graphene due to the incomplete reduction of GO in the hydrothermal reaction endows graphene excellent adsorption capacity which will help remove the organic pollutants. In this study, we prepared reduced graphene oxide (rGO)-encapsulated Fe_3_O_4_ magnetic nanospheres which were eventually anchored with TiO_2_ nanoparticles. The effects of operating parameters such as catalyst dosage and H_2_O_2_ dosage on the degradation of methylene blue (MB) were investigated. The stability of the as-prepared catalyst was also studied. The results of this study indicate that the composite catalyst possesses great promise for visible-light driven destruction of organic compounds.

## Results

### Characterization of the hybrids

**Figure 1:**
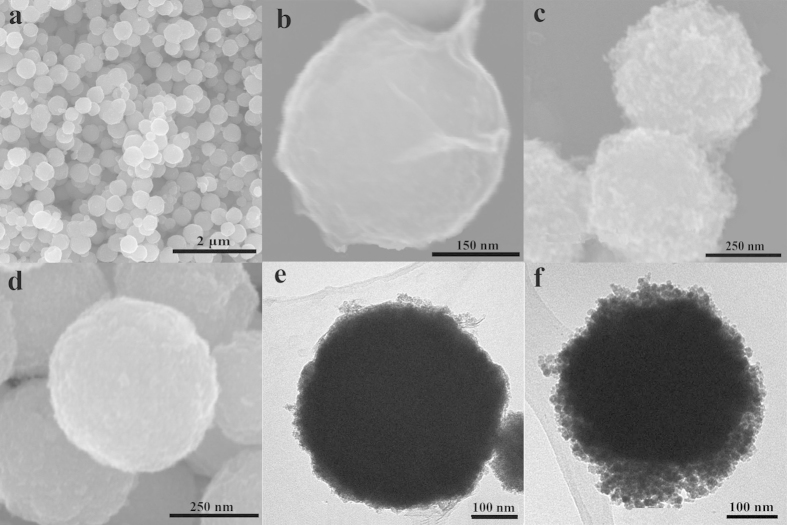
(**a**, **d**) SEM images of Fe_3_O_4_ nanoparticles prepared by solvothermal reaction. (**b**, **e**) SEM and TEM images of the GO wrapped Fe_3_O_4_ obtained by electrostatic interactions. (**c**, **f**) TEM images ofFe_3_O_4_@rGO@TiO_2_ synthesized by one-step hydrothermal reaction.

The morphology and structure of the as-prepared catalysts were characterized by SEM and TEM. As shown in Fig. 1(a, d), the Fe_3_O_4_ nanoparticles have an average diameter of about 400 ± 20 nm and the surface is rough, which can be attributed to the fact that each Fe_3_O_4_ nanospheres are composed of many smaller particles^32^. Besides, the SAED graph of Fe_3_O_4_ shown in Fig. S1a illustrates that Fe_3_O_4_ is a polycrystallinity. The 3-Aminopropyltrimethoxysilane (APTMS) molecules that reacted with Fe_3_O_4_ particles endow the magnetic spheres amino groups representing electropositive which can help the Fe_3_O_4_ nanospheres combine with the GO containing a lot of negative charged groups on its surface and edges^33^. It can be seen in Fig. 1b that there exists obvious folds on the surface of the magnetic particles which accounts for the successfully integrating GO layers with the Fe_3_O_4_ particles, corresponding perfectly to the TEM images illustrated in Fig. 1e which shows that on the surface of the Fe_3_O_4_ particles do exist thin layers. After the magnetic particles were encapsulated into the silk-like GO layers, their diameters changed a little which can be observed in Fig. 1(b, e). The SEM and TEM images shown in Fig. 1(c, f) explains that the TiO_2_ particles were successfully anchored on the GO layers according to the hydrothermal and the size of the particles increased apparently compared to Fe_3_O_4_@GO.

**Figure 2:**
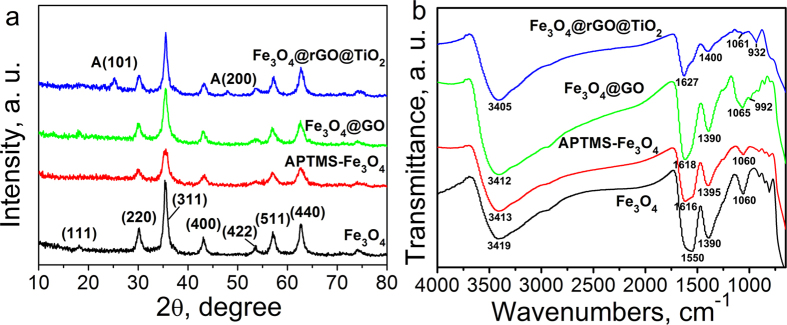
(**a**) XRD patterns and (**b**) FT-IR spectra of Fe_3_O_4_, APTMS-Fe_3_O_4_, Fe_3_O_4_@GO, Fe_3_O_4_@rGO@TiO_2_.

Figure 2a shows the XRD characterization of Fe_3_O_4_, APTMS-Fe_3_O_4_, GO-encapsulated Fe_3_O_4_ and Fe_3_O_4_@rGO@TiO_2_. As shown in Fig. 2a, characteristic diffraction peaks for Fe_3_O_4_ (2θ = 18.1°, 30°, 35.4°, 43.3°, 53.4°, 57.1° and 62.7°), which can be indexed to their indices (111), (220), (311), (400), (422), (511) and (440), were observed for the synthesized Fe_3_O_4_ nanoparticles. This result corresponds well to the PDF data (JCPDS file No. 19-0629) which shows that the magnetite diffraction peaks appeared at the same locations. Besides, this result is also in good consistence with the XRD characterization of Fe_3_O_4_ reported in the former literature^34^. After the modification by APTMS and GO, only the intensity of the obtained nanocomposites changed, which illustrated that the amount of the GO is too little to perform its crystallinity^35^. And the crystallinity of Fe_3_O_4_ modified by APTMS and GO seemed to just change a little. Two additional specific XRD diffraction peaks located at 2θ = 25.2° and 48° can be seen when the TiO_2_ nanoparticles were anchored on the surface of the GO-wrapped Fe_3_O_4_ nanospheres, which agree with the (101) and (200) planes (JCPDS file No. 21-1272) of anatase TiO_2_. Other characteristic peaks of anatase such as reported in the former literature^36^ can hardly be observed in Fig. 2a which may be attributed to the fact that these peaks may be close to the crystalline peaks of Fe_3_O_4_ that can hinder the anatase crystalline peaks.

Figure 2b shows the FT-IR spectra of Fe_3_O_4_, APTMS-Fe_3_O_4_, GO-encapsulated Fe_3_O_4_ and Fe_3_O_4_@rGO@TiO_2_. It can be clearly seen from Fig. 2b that the peaks located at 3419 and 1550 cm^−1^ are due to the H-O-H stretching and the bending vibration of the free or adsorbed water, respectively^37^. After modified by APTMS and GO, a new absorption bands situated at 1616 cm^−1^ appeared in the FT-IR spectra, which can be indexed to C = O vibration, confirming the successful wrapping of GO on the Fe_3_O_4_ nanoparticles. In comparison with the FT-IR spectra of Fe_3_O_4_@GO, the intensity of the characteristic absorption peaks of the obtained Fe_3_O_4_@rGO@TiO_2_ located at around 1627, 1400 and 1065 cm^−1^ obviously diminished which can be ascribed to the reduction of GO. However, the absorption peaks located at around 1627, 1400 and 1065 cm^−1^ still existed, illustrating that the GO was not completely reduced. Moreover, the Raman spectrum shown in Fig. S2 can also account for the successful combination of Fe_3_O_4_, rGO and TiO_2_.

**Figure 3:**
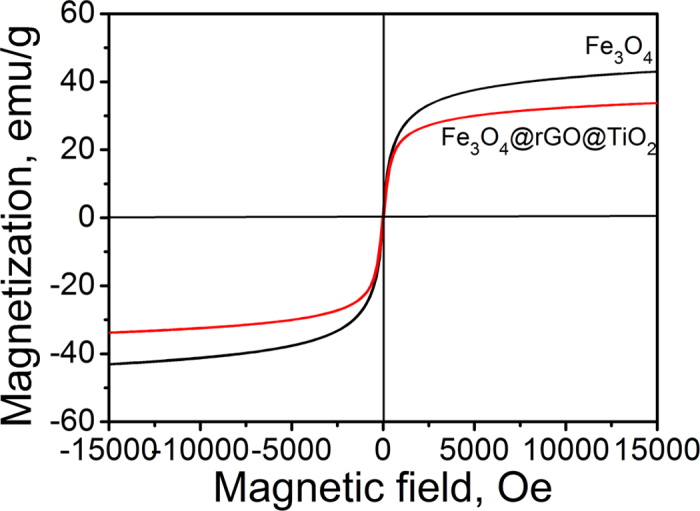
Room temperature magnetic hysteresis loops of Fe_3_O_4_ and Fe_3_O_4_@rGO@TiO_2_.

The magnetic properties of the as-prepared Fe_3_O_4_@rGO@TiO_2_ were tested by using a vibrating sample magnetometer at room temperature. As can been seen in Fig. 3, the saturation magnetization (Ms) value of Fe_3_O_4_ was 43.691 emu/g, while for Fe_3_O_4_@rGO@TiO_2_ it was just 34.202 emu/g, mainly due to the existence of the rGO wrapped on the surface of Fe_3_O_4_ and the subsequently anchored TiO_2_ nanoparticles^38^. The obtained catalysts can be quickly separated from solution under an external magnetic field because of their considerable Ms values, which will be beneficial for their reuse and boosting the overall water treatment efficiency in practical applications.

### Photo-Fenton degradation activities of Fe_3_O_4_@rGO@TiO_2_

MB was chosen as the model organic pollutant to evaluate the degradation activities of the as-prepared hybrids. The concentration of MB was monitored by measuring the absorbance at a wavelength of 664 nm characteristic of MB. The suspension composed of MB and Fe_3_O_4_@rGO@TiO_2_ were stirred in the dark for about 30 min to achieve absorption-desorption equilibrium. The concentration of the MB was regarded as the initial concentration C_0_. Apparent degradation of MB was observed as soon as the photo-Fenton reaction was initiated by introducing illumination as well as H_2_O_2_ to the system.

**Figure 4:**
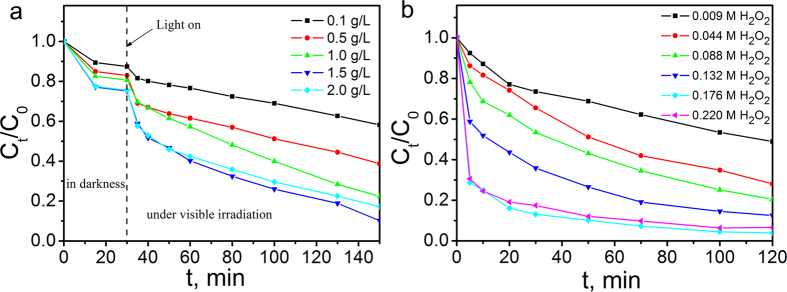
Photo-Fenton degradation of MB in the presence of Fe_3_O_4_@rGO@TiO_2_ at room temperature under neutral pH. (**a**) Effect of catalyst dosage on MB degradation (initial MB concentration, 10 mg/L; H_2_O_2_, 0.088 M); (**b**) Effect of H_2_O_2_ dosage on MB degradation(initial MB concentration, 10 mg/L; catalysts, 1.5 g/L).

The effect of the dose of the catalysts on the degradation activity in the Fenton process was illustrated in Fig. 4a. It can be concluded that the degradation accelerated as the amount of the catalyst increased from 0.1 to 1.5 g/L, but dropped with excessive dosage, as can be seen from the data of dosage of 2.0 g/L. This phenomenon can be ascribed to the reason that the number of the reactive sites can be increased when the amount of the composites were increasing. However, these nanoparticles may have a tendency to aggregate when their quantity is in excess, thus contributing to the decrease of the reactive sites. Besides, excess amount of Fe_3_O_4_ may exist as the scavenger of hydroxyl radicals^38^,^39^,^40^,^41^. In this study, the optimal amount of the catalyst was 1.5 g/L just as shown in Fig. 4a.

The effect of amount of H_2_O_2_ on the degradation of MB in the Fenton-like system was also investigated in this work. The result illustrated in Fig. 4b clearly shows enhanced degradation activity as the amount of H_2_O_2_ increased, but demonstrates saturation and slight decrease when the concentration is beyond 0.176 M. As is well known that the generation rate of hydroxyl radicals (·OH) can be accelerated as more H_2_O_2_ is introduced to the Fenton system at the beginning, which is beneficial to the degradation of the organic dyes. Nevertheless, when the amount of H_2_O_2_ is achieving a critical point, the generated hydroxyl radicals (·OH) may react with the excessive H_2_O_2_ which is not initiated by the catalysts in time^39^,^40^. Therefore, the catalytic performance can only achieve a maximum effect when the utilization of H_2_O_2_ is optimal. This theory was totally accorded with the consequence illustrated in Fig. 4b. When the amount of H_2_O_2_ was over 0.176 M, it was distinctly seen that the degradation activity slightly decreased. To verify that the great degradation efficiency is due to the existence of the catalysts, different amount of H_2_O_2_ was used to remove MB under the same condition without any catalysts. The results are depicted in Fig. S3, showing that H_2_O_2_ just have slight effect on the degradation of MB.

### Stability of Fe_3_O_4_@rGO@TiO_2_

**Figure 5:**
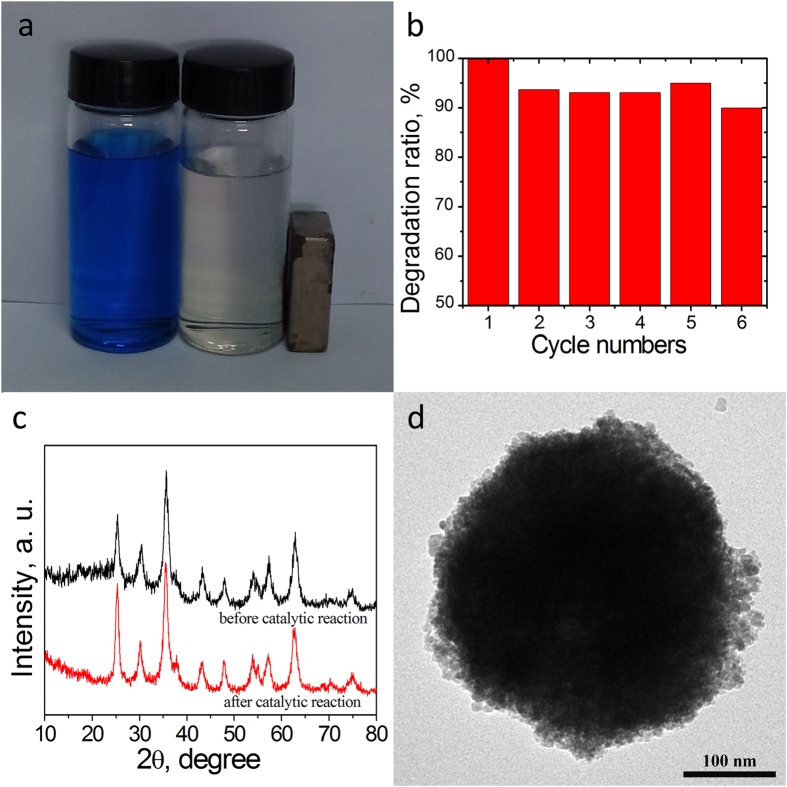
(**a**) Photograph of MB before and after photo-Fenton reaction. (**b**) The cyclic utilization of the as-prepared Fe_3_O_4_@rGO@TiO_2_ hybrids for the degradation of MB with the addition of H_2_O_2_ and illumination at neutral pH and room temperature for 120 min. (**c**) XRD patterns of Fe_3_O_4_@rGO@TiO_2_ before and after six cycles. (**d**) TEM image of Fe_3_O_4_@rGO@TiO_2_ after six cycles.

The stability of the catalytic materials is of great importance if the catalysts are to be practically applicable. The as-prepared catalyst Fe_3_O_4_@rGO@TiO_2_ can be easily separated from the MB solution by magnetic field and for reuse. Figure 5a shows the photograph comparing the MB solutions before and after the Fenton reaction, from which we can distinctly observe that MB almost totally discolored after the reaction. As depicted in Fig. 5b, the removal of MB during the first catalytic run could be achieved above 99.0% after 2 h. After six recycles for the catalytic degradation of MB, the catalytic activity of Fe_3_O_4_@rGO@TiO_2_ just slightly decreased. As can be seen in Fig. 5b, the degradation efficiency of Fe_3_O_4_@rGO@TiO_2_ can reach up to 93% after 2 h even if the catalysts had been utilized for several times, which indicates that this catalyst can maintain good stability. The TEM image of the Fe_3_O_4_@rGO@TiO_2_ which had been used for six times shown in Fig. 5d also proved the catalysts’ stability. The morphology of the as-prepared catalyst did not undergo obvious change even after several cycles. The XRD patterns (Fig. 5c) of the freshly prepared catalyst and the catalyst recycled after many times further illustrated the stability of the catalysts.

## Discussion

**Figure 6:**
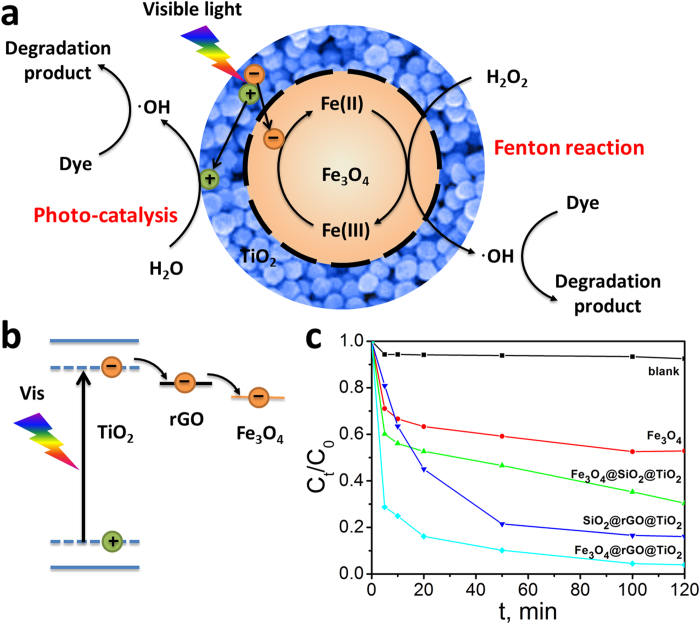
(**a**, **b**) Suggested mechanism for the photo-Fenton degradation of MB by Fe_3_O_4_@rGO@TiO_2_ at room temperature and neutral pH. (**c**) Photo-Fenton degradation of MB at room temperature and neutral pH by different catalysts (blank, Fe_3_O_4_, Fe_3_O_4_@SiO_2_@TiO_2_, SiO_2_@rGO@TiO_2_ and Fe_3_O_4_@rGO@TiO_2_).

One essential concern on the present Fenton system is that which component has the main effect on decomposition of H_2_O_2_ into hydroxyl radicals. To verify the contributions from different components to the degradation of MB, several different materials including Fe_3_O_4_, Fe_3_O_4_@SiO_2_@TiO_2_, SiO_2_@rGO@TiO_2_ and Fe_3_O_4_@rGO@TiO_2_, were chosen to act as the catalysts. The degradation processes under different conditions are shown in Fig. 6c. It can be obviously seen from the five degradation curves that Fe_3_O_4_@rGO@TiO_2_ shows the best degradation performance. The degradation process was quick at first when Fe_3_O_4_ was used as the catalyst. However, the reaction rate decreased after 10 min, which can be attributed to the reason that the ferrous ions existed in Fe_3_O_4_ had been almost totally consumed at beginning and the conversion rate of ferric to ferrous is far less than the ferric ions’ consumption rate^36^,^37^,^41^,^42^. It can also draw a conclusion from Fig. 6c that when SiO_2_@rGO@TiO_2_ or Fe_3_O_4_@SiO_2_@TiO_2_, whose structure and morphology is similar to Fe_3_O_4_@rGO@TiO_2_ except for the component, were utilized as the catalysts to decompose MB, their performance is not as efficient as Fe_3_O_4_@rGO@TiO_2_. It may be ascribed to the synergic effect between the three different components. The assumed mechanism can be summarized into three aspects and has been illustrated in Fig. 6(a, b). (1) Fe_3_O_4_, the core of Fe_3_O_4_@rGO@TiO_2_, is the major component used to react with H_2_O_2_ to generate hydroxyl radicals (•OH) to finally decompose MB. Besides, the ferromagnetic of Fe_3_O_4_ make the catalysts facilely separable from the solution for subsequent usage. (2) The existence of rGO not only endows the catalysts good adsorption of MB which may be beneficial to the degradation of MB, but also endues the catalysts with extended usage of the solar energy. Besides, the band gap of TiO_2_ can be narrowed from 3.2 to 2.8 eV when TiO_2_ was combined with GO^20^ (see supplementary information Fig. S4 for certification) so that the valence electrons of TiO_2_ can be also excited to the conduction band state only under visible light irradiation^24^,^25^,^26^,^27^,^28^,^29^,^30^,^31^. (3) The presence of TiO_2_ may also have a great effect on the degradation of MB. When irradiated by the visible light (*λ* > 400 nm), TiO_2_ anchored on GO can be excited to generate photo-induced electrons and holes, and the existence of GO may be able to transport the photo-induced electrons^43^ to inner Fe^3+^ to help the Fe^3+^ to be reduced to Fe^2+^. Furthermore, the rapid transfer rate of the photo-generated electrons from TiO_2_ to Fe^3+^ prolong the life time of the photo-generated holes whose oxidative potential is also high enough to degrade most organic pollutants. The opportune isolation of the photo-generated electrons and holes effectively avoids their recombination so that the residue holes may be able to straightly react with the organic pollutants to make the organics degrade^44^. All the advantages brought by these components make the catalysts composed of Fe_3_O_4_, GO and TiO_2_ perform better catalytic activity compared with the bare Fe_3_O_4,_ SiO_2_@rGO@TiO_2_ and Fe_3_O_4_@SiO_2_@TiO_2_ nanoparticles, illustrating that the catalysts cannot manifest good degradation efficiency unless they hold all of the three components.

In summary, the sphere-like ternary Fe_3_O_4_@rGO@TiO_2_ as an efficient heterogeneous Fenton-like catalyst was successfully synthesized in our work. The GO was wrapped on Fe_3_O_4_ nanospheres by using an electrostatic layer-by-layer method. The TiO_2_ nanoparticles were anchored on the surface of Fe_3_O_4_@GO and the GO was incompletely reduced to rGO through the hydrothermal reaction simultaneously. The experiments of degrading MB confirmed that the obtained catalysts can perform great catalytic activity even if it was used in the neutral pH and irradiated by visible light. And the catalysts could still exhibit high catalytic activity even though it had been used for many times. The comparison of the catalytic activity among Fe_3_O_4_, Fe_3_O_4_@SiO_2_@TiO_2_, SiO_2_@rGO@TiO_2_ and Fe_3_O_4_@rGO@TiO_2_ showed that the three components composed the catalysts possessed synergic effects which may be beneficial to the degradation of the recalcitrant organics.

## Methods

### Materials and preparation

All other chemical reagents were purchased from Shanghai Chemical Reagent Co. All chemicals were used without any purification. Ultrapure water (18 MUcm) was used for all experiments.

Graphene oxide (GO) was prepared from natural graphite by a modified Hummers method^45^. In a typical procedure, 2 g natural flake graphite, 2 g of NaNO_3_, together with 96 mL of concentrated H_2_SO_4_ were mixed at 0 °C. Then, 12 g of KMnO_4_ was gradually added to the obtained mixture and continuously stirred for 90 min while keeping the temperature at 0 °C. Thereafter, the mixture was heated to 50 °C and stirred for 2 h. 30 mL of concentrated HNO_3_ was slowly added to the above mixture and keeping stirring at 50 °C for about 2 h. Then, distilled water (80 mL) was slowly dropped into the resulting solution to dilute the mixture, and the stirring continued for 1 h at 95 °C. Finally, 10 mL of H_2_O_2_ (30%) were added to react with the residual KMnO_4_. The graphite oxide deposit was collected from the graphite oxide suspension by centrifugation at 10000 rpm for 15 min, and washed with distilled water for several times to remove the residual ions. Then the obtained graphene oxide suspension was dialyzed for two weeks to get the final products.

### Preparation of GO encapsulated Fe_3_O_4_

**Figure 7:**
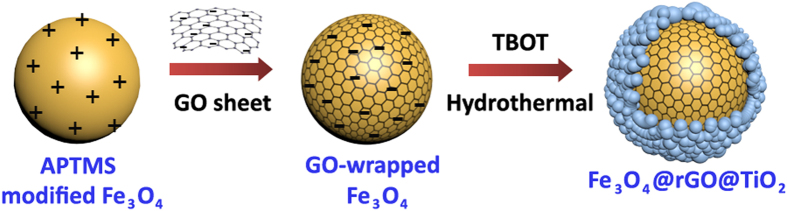
Schematic illustration of synthesis steps for Fe_3_O_4_@rGO@TiO_2_ hybrid. (**a**) Fe_3_O_4_ modified by APTMS. (**b**) Synthesis step of GO wrapped Fe_3_O_4_. The hybrid was synthesized through electrostatic interactions. (**c**) Synthesis step of Fe_3_O_4_@rGO@TiO_2._The hybrid was synthesized through one step hydrothermal GO reduction and TiO_2_ crystallization.

1.299 g of FeCl_3_, 0.5 g of trisodium citrate, and 2.0 g of NaAc were dissolved in 40 mL of ethylene glycol with magnetic stirring. The homogeneous yellow solution was then transformed into a 100 mL Teflon-lined stainless-steel autoclave, heated at 200 °C for about 10 h, and then cooled to room temperature. The obtained black products were washed by ethanol and distilled water for three times, respectively. 0.5 g of the obtained Fe_3_O_4_ was homogeneous dispersed in isopropyl alcohol solution by ultrasonic for 30 min. Afterwards, 0.5 mL of APTMS were added to the above mixture and refluxed at 80 °C for 24 h. The products shown in Fig. 7a were washing by ethanol for several times and then dried in a vacuum oven. Finally, 100 mL homogeneous aqueous solution of the APTMS modified Fe_3_O_4_ (APTMS-Fe_3_O_4_) was mixed with 150 mL of 0.5 mg/mL GO for about 30 min under mechanical stirring to get GO wrapped Fe_3_O_4_ (Fe_3_O_4_@GO) nanospheres just like the image illustrated in Fig. 7b.

### Synthesis of Fe_3_O_4_@rGO@TiO_2_

30 mg of the as-prepared Fe_3_O_4_@GO were dispersed in 25 mL isopropyl alcohol by ultrasonic for 30 min. 100 μL tetrabutyl titanate (TBOT) were slowly dropped into the mixture and continuously stirred for another 30 min. Then 1 mL distilled water was added dropwise into the above solution. After keeping stirring for 30 min, the mixture was transformed into a 50 mL Teflon-lined stainless-steel autoclave, heated at 180 °C for 8 h, then cooled to temperature. The end products shown in Fig. 7c were rinsed by ethanol and distilled water for three times, respectively. After dried at 70 °C under vacuum, a grizzly ternary composite with TiO_2_ nanoparticles decorating on the surface of the GO-wrapped Fe_3_O_4_ sphere was obtained.

### Degradation of MB by heterogeneous photo-Fenton reaction

The photo-Fenton activity of the as-prepared catalysts was evaluated by photodegradation of MB in aqueous solution under visible irradiation. A 300 W UV-vis lamp equipped with a λ > 400 nm cut off filter which covered the window of the Xenon lamp to absorb UV light and allow visible light to pass through was used as a light source^46^. All catalytic reactions were conducted in a 100 mL radius flask with constant mechanical agitation at room temperature. For the degradation of MB, desired amount of the as-prepared catalysts were added into the 50 mL aqueous solution containing 10 mg/L MB. Before illumination, the suspension without any H_2_O_2_ was sufficiently stirred for 30 min to reach adsorption-desorption equilibrium between the catalysts and MB so that the adsorption in the dark can be discounted. The lamp was turned on while a certain amount of H_2_O_2_ was adding to the mixed solution. About 5 mL aliquots were withdrawn at given time intervals and the catalysts were collected by magnetic separation. The concentration of the remnant MB was determined by testing the absorbance of the supertant at 664 nm by UV-vis spectroscopy.

### Characterizations

To demonstrate the surface morphology and structure of the as-prepared catalysts, the samples were examined by scanning electron microscopy (SEM) using a JEOL SM-6360LV microscope equipped with an energy dispersive X-ray analyzer (EDX). Transmission electron microscopy (TEM) observation was achieved with a JEOL 2011 microscope (Japan) operated at an acceleration voltage of 200 kV. All the samples were suspended in the anhydrous ethyl alcohol and dropped on a carbon-coated copper grid, followed by drying at room temperature overnight. Power X-ray diffraction (XRD) measurements was performed on a X-ray diffractometer (RIGAKU, D/MAX 2550VB/PC, Japan) with CuKα radiation to verify the crystalline structure of the catalysts. The UV-vis spectra were recorded on a UV-vis spectrometer (UNICO UV-2102PC) at room temperature. The magnetization curve of the product was measured with a vibrating sample magnetometer (LAKE SHORE, 7407).

## Author Contributions

X.L.Y. and W.C. conceived and designed the experiments. W.C. analyzed results and wrote the manuscript. Y.H.Z. and J.F.H. advised W.C. and reviewed the manuscript. Y.Z. and C.Z.L. reviewed the manuscript. All authors discussed the results and commented on the manuscript.

## Additional Information

**How to cite this article**: Yang, X. *et al.* Rapid degradation of methylene blue in a novel heterogeneous Fe_3_O_4_@rGO@TiO_2_-catalyzed photo-Fenton system. *Sci. Rep.* **5**, 10632; doi: 10.1038/srep10632 (2015).

## Supplementary Material

Supporting Information Supplementary Figures 1-6
