# Supplementary material for: Rapid degradation of methylene blue in a novel heterogeneous Fe3O4 @rGO@TiO2-catalyzed photo-Fenton system
Source: Sci Rep. 2015 May 22;5:10632. doi: 10.1038/srep10632 (PMC4441169; doi:10.1038/srep10632)
Supplement: Supporting Information — Supplementary Figures 1-6 [file srep10632-s1.pdf]

Title:

# Rapid degradation of methylene blue in a novel heterogeneous $Fe_3O_4$@rGO@$TiO_2$-catalyzed photo-Fenton system

Author names

Xiaoling Yang, Wei Chen, Jianfei Huang, Ying Zhou, Yihua Zhu,* and Chunzhong Li

Affiliations

East China University of Science and Technology, Key Laboratory for Ultrafine Materials of Ministry of Education, School of Materials Science and Engineering, Shanghai 200237, China

* Authors to whom correspondence should be addressed

yhzhu@ecust.edu.cn

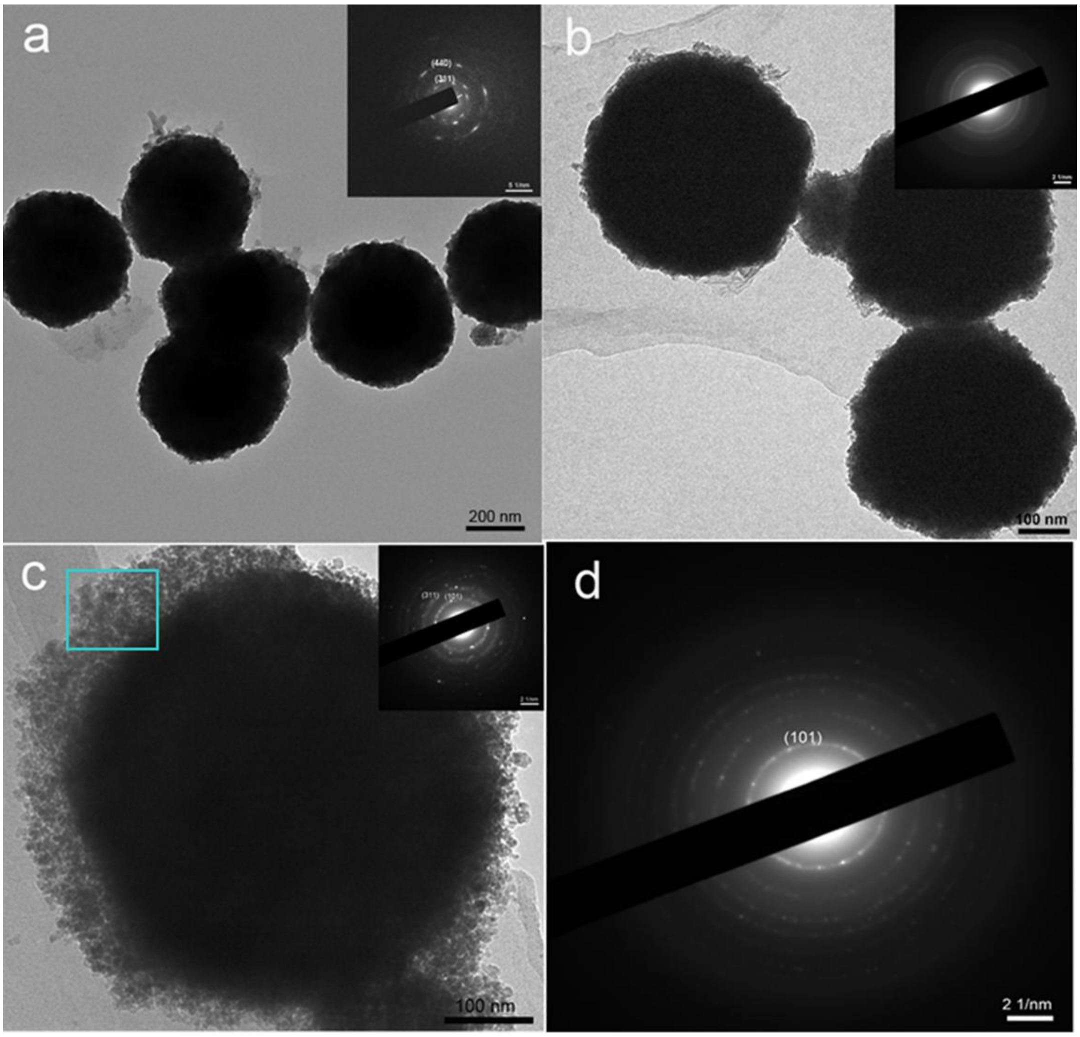


Fig. S1 (a-c) HRTEM and selected area electron diffraction (SAED) patterns of $Fe_3O_4$, $Fe_3O_4$@GO and $Fe_3O_4$@rGO@$TiO_2$; (d) SAED image of the selected area in Fig. S1c.

The SAED image of $Fe_3O_4$ particles shown as an inset of Fig. S1a illustrates that $Fe_3O_4$ is a polycrystallinity. The calculated lattice spacings from the diffraction patterns, with the values of 0.246 and 0.143 nm, match perfectly with that of (311) and (440) planes of $Fe_3O_4$ (JCPDS file No. 19-0629). The SAED pattern of $Fe_3O_4$@GO (Figure. S1b) shows only diffraction rings without any diffraction dots, which may be attributed to the existence of the amorphous GO [1,2]. Moreover, the HRTEM and SAED graph of $Fe_3O_4$@rGO@$TiO_2$ composites illustrated in Fig. S1c

revealed that $TiO_2$ nanospheres were successfully deposited on the surface of the GO-wrapped $Fe_3O_4$ particles. Another electron diffraction image of the selected area in Figure. S1c with distinct diffraction rings was depicted in Figure. S1d. The lattice spacing calculated from the diffraction rings agrees well with that of (101) planes of $TiO_2$ (JCPDS file No. 21-1272), indicating that anatase $TiO_2$ was grown in $Fe_3O_4$@rGO@$TiO_2$ successfully and $TiO_2$ existed in the composites is also a polycrystallinity.

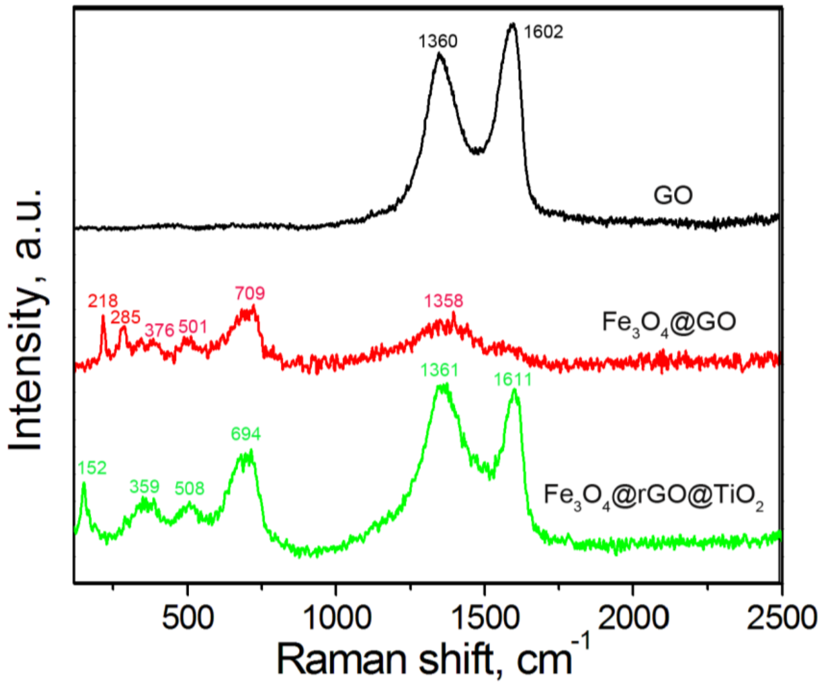


Fig. S2 Raman spectra of GO, $Fe_3O_4$@GO, $Fe_3O_4$@rGO@$TiO_2$.

Fig. S2 shows the Raman spectrogram of GO, $Fe_3O_4$@GO and $Fe_3O_4$@rGO@$TiO_2$. As can be seen in the above graph, GO shows an obvious Raman shift at 1360 and 1602 $cm^{-1}$ corresponding to the D- and G- bands, respectively [3]. The Raman signals located at 218, 285, 376, 501 and 709 $cm^{-1}$, corresponding to the vibration modes of Fe-O bonds of $Fe_3O_4$ nanoparticles could be observed in

$Fe_3O_4$@GO [4]. The reason that the D- and G- bands of GO centered at 1358 $cm^{-1}$ in $Fe_3O_4$@GO may be due to overlap of two peaks, one of $Fe_3O_4$ at 1300 $cm^{-1}$ [5] and a GO D band peak at 1360 $cm^{-1}$. Some new peaks occurred at 152, 359, 508, 694 $cm^{-1}$ when $TiO_2$ nanoparticles were decorated on the surface of the GO-wrapped $Fe_3O_4$, all of which are characteristic peaks of anatase $TiO_2$ [6]. Besides, the intensity ratio of D- and G- bands of GO ($I_D/I_G$) changed after depositing $TiO_2$ nanoparticles on the surface of the GO-encapsulated $Fe_3O_4$ nanospheres through the hydrothermal reaction, indicating that the structure defects increased after GO was transformed into rGO partially. The raman spectrum of $Fe_3O_4$@rGO@$TiO_2$ is actually a simple combination of that of $Fe_3O_4$, rGO and $TiO_2$. This illustrates that the combination of the three components is just a physical force.

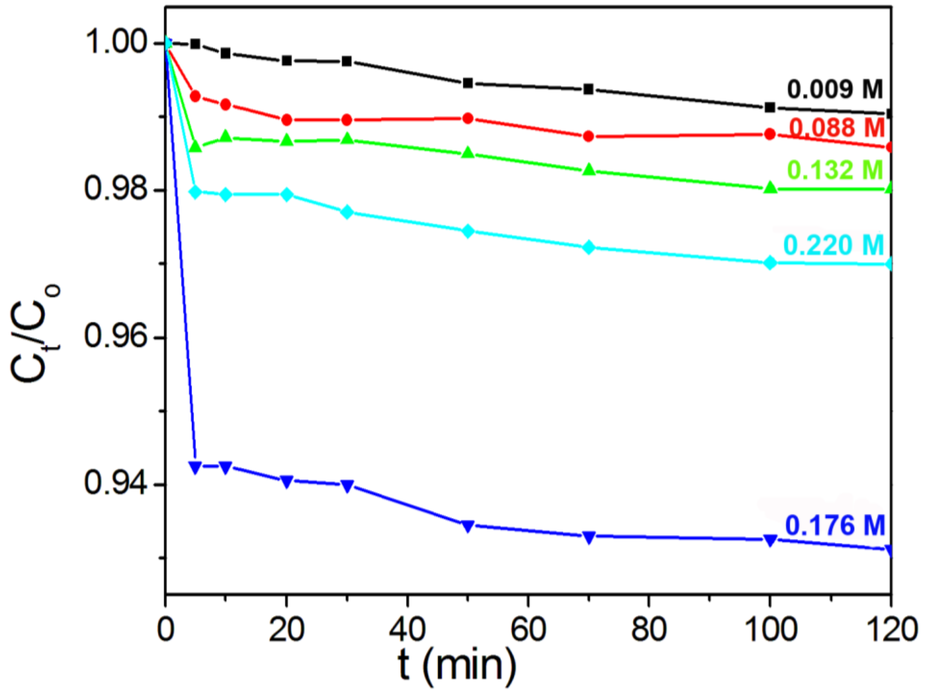


Fig. S3 Effect of dose of $H_2O_2$ on the degradation of MB without any catalysts at room temperature and neutral pH.

As can be seen in the Fig. S3, different dose of $H_2O_2$ just have slight effect on the degradation of MB and the degradation tendency is similar to the consequence shown in Fig 4b. These results further confirm that the as-prepared catalysts do have a great effect on the degradation of MB, and the degradation efficiency can increase as the amount of $H_2O_2$ increasing until an optimal point, and a slight decreasing will occur when the amount of $H_2O_2$ is beyond the optimum which can be attributed to the side reaction between $H_2O_2$ and ·OH.

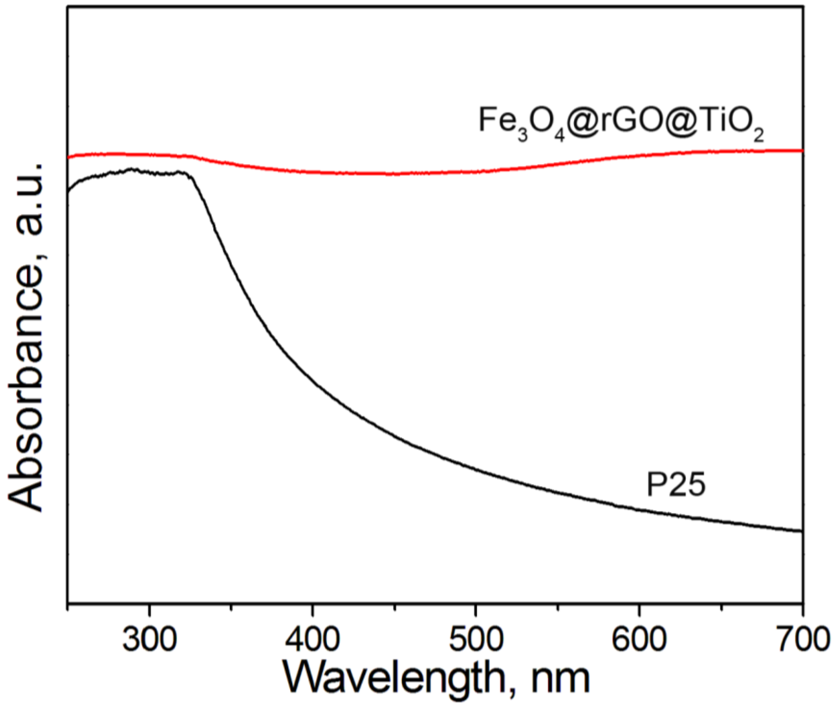


Fig. S4 UV-visible spectra of P25 and $Fe_3O_4$@rGO@$TiO_2$

It can be observed from Fig. S4 that $Fe_3O_4$@rGO@$TiO_2$ has a more intensive absorption of visible light compared with P25 due to coexistence of rGO and $Fe_3O_4$. Besides, a red shift in the absorption edge is clearly presented, illustrating that the band gap of $TiO_2$ has been narrowed and the electrons in the valance band can be excited to the conduction band under the visible illumination.

[1] Xie, G. Q. *et al.* A facile chemical method to produce superparamagnetic graphene oxide-$Fe_3O_4$ hybrid composite and its application in the removal of dyes from aqueous solution. *J. Mater. Chem.* **22,** 1033-1039 (2012).

[2] Wang, G. X. *et al.* Facile synthesis and characterization of graphene nanosheets. *J. Phys. Chem. C* **112,** 8192-8195 (2008).

[3] Sher Shah, M. S. A., Park, A. R., Zhang, K., Park, J. H., Yoo, P. J. Green synthesis of biphasic $TiO_2$-reduced graphene oxide nanocomposites with highly enhanced photocatalytic activity. *ACS Appl. Mater. Interfaces* **4,** 3893-3901 (2012).

[4] Hu, X. B. *et al.* Adsorption and heterogeneous Fenton degradation of 17α-methyltestosterone on nano $Fe_3O_4$/MWCNTs in aqueous solution. *Appl. Catal., B* **107,** 274-283 (2011).

[5] Wang, J. Z. *et al.* Graphene - encapsulated $Fe_3O_4$ nanoparticles with 3D laminated structure as superior anode in lithium ion batteries. *Chem. Eur. J.* **17,** 661-667 (2011).

[6] Jang, B. J. *et al.* In situ growth of $TiO_2$ in interlayers of expanded graphite for the fabrication of $TiO_2$-graphene with enhanced photocatalytic activity. *Chem. Eur. J.* **17,** 8379-8387 (2011).
